# Adsorption of Crystal Violet onto an Agricultural Waste Residue: Kinetics, Isotherm, Thermodynamics, and Mechanism of Adsorption

**DOI:** 10.1155/2020/5873521

**Published:** 2020-05-01

**Authors:** Ilyasse Loulidi, Fatima Boukhlifi, Mbarka Ouchabi, Abdelouahed Amar, Maria Jabri, Abderahim Kali, Salma Chraibi, Chaimaa Hadey, Faissal Aziz

**Affiliations:** ^1^Laboratory of Chemistry and Biology Applied to the Environment, Faculty of Sciences, Moulay Ismail University, BP 11201-Zitoune, Meknes, Morocco; ^2^Laboratory of Catalysis and Corrosion of Materials, Chouaïb Doukkali University, Faculty of Sciences El Jadida, BP. 20, El Jadida, Morocco; ^3^Engineering Sciences and Trades Laboratory, ENSAM, University Moulay Ismail, Meknes, Morocco; ^4^National Centre for Research and Study on Water and Energy (CNEREE), Cadi Ayyad University, Marrakech, Morocco

## Abstract

Agricultural waste can be exploited for the adsorption of dyes, due to their low cost, availability, cost-effectiveness, and efficiency. In this study, we were interested in the elimination of crystal violet dye, from aqueous solutions, by adsorption on almond shell-based material, as a low-cost and ecofriendly adsorbent. The almond shells were first analyzed by Fourier transform infrared spectroscopy (FTIR) and X-ray diffraction; then, the influence of adsorbent dose, initial dye concentration time, and pH were studied to assess adsorption capacity under optimal experimental conditions. Experimental results indicate that almond shell adsorbent removes about 83% of the dye from the solutions at room temperature and in batch mode; the kinetic study showed that the equilibrium time is about 90 min, and the model of pseudo-second order could very well describe adsorption kinetics. The modulation of adsorption isotherms showed that retention follows the Langmuir model. The thermodynamic study has shown that the adsorption is endothermic (Δ*H*° > 0) and spontaneous (Δ*G*° < 0).

## 1. Introduction

Many industries, such as the textile, plastics, and paper, use dyes to hue their products, and large volumes of water are consumed. Consequently, they generate a considerable quantity of colored wastewaters. More than 100000 dyes available in the trade with more than 7 10^5^ t of dyes are produced annually in the world [1]. 1000 t of these dyes are released annually into the aquatic system [2].

The dye is the first pollutant to be detected in wastewater [3]. The presence of a very low concentration of dyes, in water, is very noticeable and undesirable. Several treatment methods were used for treating effluents containing dyes [2, 4]. But these differ in their effectiveness, cost, and environmental impact [5]. Adsorption is the most effective technique widely used [6, 7], in which activated carbon is the most frequently used adsorbent in the purification of water. The high cost required by adsorption using activated carbon [2] pushes researchers to find other alternatives such as waste or agricultural by-products [4]. Agricultural waste consists mainly of cellulose, hemicellulose, and lignin, which are effective adsorbents for a wide range of pollutants because of their richness in functional groups such as hydroxyl groups, carboxyl, and phenols [8]. Other advantages that make them as excellent candidates are the capacity and the rate of adsorption, the high selectivity for different pollutants, and also rapid kinetics [4]. Agricultural waste is better than other adsorbents, as they are generally used without or with minimal treatment (washing, drying, and grinding) [9]. Several recent studies have used various agricultural wastes for adsorption of dyes in effluents.  Table 1 shows some examples.

Almond is the fruit of almond tree, which is the second most important fruit species cultivated in Morocco after the olive tree, with a production of 101,000 t/year in 2016, which is the equivalent of 80,000 tonnes of almond shells, according to data from the Moroccan Federation of Almond Tree Producers. These residues are discarded as solid waste, which poses environmental problems. It is therefore necessary to find an appropriate method to solve the disposal problem. The use of this inexpensive material as an adsorbent contributes to the solution to this problem and to the application of the principle of “self-cleaning of waste.”

Crystal violet is widely used as a violet dye in the textile industry for dyeing cotton and silk. It is also used in the manufacture of paints and printing inks [10]. Crystal violet is carcinogenic and has been classified as a recalcitrant molecule because it is poorly metabolized by microbes, is nonbiodegradable, and can persist in various environments. It is highly toxic to the cells [11].

The purpose of this research paper is to investigate the potential use of almond shells to remove the crystal violet dye from aqueous solutions. Adsorption parameters including dye concentration, contact time, almond shell dose, and pH were studied to determine the effectiveness of the adsorbent. The characterization of the adsorbent before and after adsorption was achieved in order to identify the mechanism governing the fixation of the dye molecules on the adsorbent.

## 2. Materials and Methods

### 2.1. Adsorbent Preparation

The almond shells (AS) were washed, dried for 24 hours at a temperature of 110°C in an oven, and then crushed and sieved to obtain fine and homogeneous samples (<0.2 mm).

### 2.2. Adsorbate Preparation

Crystal violet (CV) (characteristics given in Table 2) dye was used as the adsorbate. A stock solution of crystal violet (1 g/L) was first prepared by dissolving a known quantity in deionized water. The stock solution was finally diluted to obtain the desired concentration.

### 2.3. Adsorption Study

All adsorption experiments were realized at room temperature (≈25°C) and in the batch mode. A mass of the adsorbent was contacted with a volume *V* = 40 ml of the initial crystal violet solution *C*_0_. The assembly was agitated for a time *t* of adsorption, and then, the solid was separated from the solution by filtration on a microporous filter. The absorbance of the supernatant solution was measured using a UV-vis spectrophotometer at the wavelength corresponding to the maximum absorbance (*λ*_max_ = 590 nm). The concentration at time *t* (*C*_*t*_) of the dye in the mixture was calculated using a calibration curve prepared from the known concentrations of the CV. The removal percentage (*R*_*t*_ (%)) of the CV and the quantity adsorbed to the surface of the AS (*q*_*t*_ (mg/g)) were determined using the following equations [23]:

(1) $$\begin{array}{c} R_{t}=\frac{\left(C_{0}-C_{t}\right)\cdot 100}{C_{0}}, \end{array}$$

(2) $$\begin{array}{c} q_{t}=\frac{\left(C_{0}-C_{t}\right)\cdot V}{m}, \end{array}$$

where *C*_0_ and *C*_*t*_ are the CV initial and final concentration (mg/L) at time *t*, *V* is the solution volume (L), and *m* is the adsorbent mass (g).

## 3. Results and Discussion

### 3.1. X-Ray Diffraction Analysis

**Figure 1:**
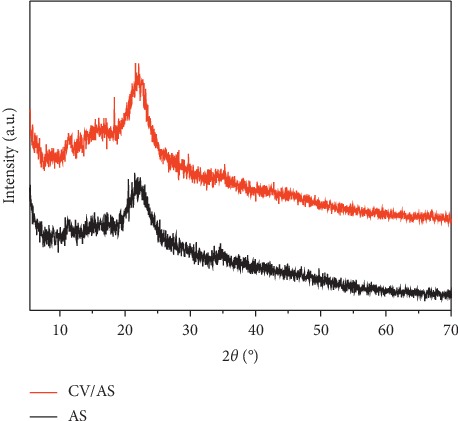
X-ray diffraction spectra of almond shell before (AS) and after adsorption (CV/AS).

Figure 1 shows the diffractograms of the AS before and after adsorption of the dye. It can be seen on the one hand that the two diffractograms are identical which means that the material undergoes no modification after adsorption, and on the other hand, the broadband at about 22° justifies certain crystalline phases in the material. In fact, lignocellulosic materials present defects of structures offering the possibility of obtaining monocrystals called whiskers [24].

### 3.2. Fourier Transform Infrared (FTIR) Spectroscopy

**Figure 2:**
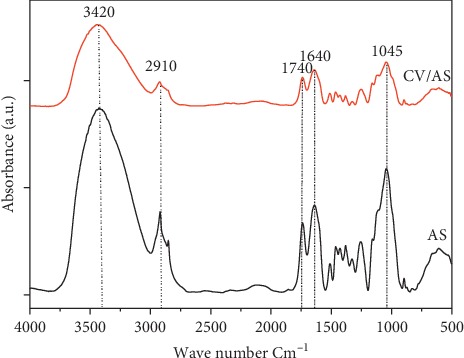
FTIR spectra of almond shell before (AS) and after CV adsorption (CV/AS).

FTIR spectroscopy is a widely used method for determining the functional groups that serve as adsorption sites.  Figure 2 shows the FTIR spectra of the AS before and after CV adsorption. The analysis of the FTIR spectrum shows the presence of many peaks in the range of wavenumbers from 4,000 to 500 cm^−1^, which highlights the complex nature of the material analyzed. Before adsorption, the broad band at about 3420 cm^−1^ corresponds to the elongation of the O-H groups, the band at 2910 cm^−1^ relates to the elongation of the C-H group, and the band at 1740 corresponds to the elongation vibration of the nonconjugated C=O bonds; these vibrations are mainly due to the ester and carboxylic acid functions present in the lignin, pectin, and hemicelluloses; the 1640 cm^−1^ band is characteristic for the elongation of the C=C bonds of aromatic compounds, and the 1045 cm^−1^ band is characteristic of the deformation in the C-O plane of aromatic compounds and acetyl and carboxylic acid functions. After adsorption of the dye, the intensity of the bands decreased significantly and the band of elongation of the O-H widens, indicating the presence of interactions between the AS and CV functional groups.

### 3.3. Effect of Physicochemical Parameters on Dye Removal Efficiency

#### 3.3.1. Effect of Contact Time

**Figure 3:**
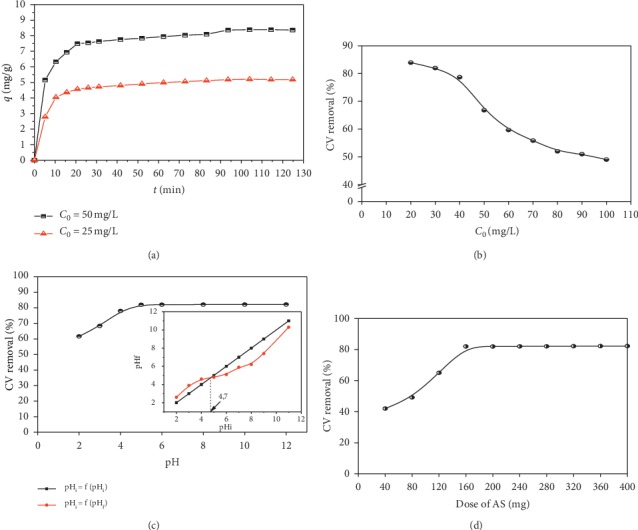
Effect of (a) contact time on adsorption; (b) initial dye concentration on adsorption; (c) pH on CV adsorption; (d) the adsorbent dose on adsorption process. The inset represents the pH variation in terms of the initial pH of the solution to determine the point of zero charge pH_pzc_.

It is necessary to obtain the time at the end of which the adsorption equilibrium is reached. This study was conducted for concentrations of 25 mg/L and 50 mg/L. The results obtained are shown in Figure 3(a), which illustrate the evolution of the adsorbed quantity over time. From the figure, we can see that the equilibrium is reached almost at the end of 90 minutes. The results show the existence of two phases: the first rapid and the second slow. This is related to the high availability of the adsorbent-free active sites at the beginning of the experiment, which decrease as the adsorption progresses. These curves also show that the fixed quantity *q*_*t*_ increases with *C*_0_. The curve of *C*_0_ = 25 mg/L is lower than that of *C*_0_ = 50 mg/L.

#### 3.3.2. Effect of Initial Crystal Violet Concentration

For this study, the initial concentration was varied in the range of 20 mg/L to 100 mg/L by maintaining the adsorbent dose at 5 g/L, the temperature at 20°C, and the pH at 6. The results are shown in Figure 3(b). It can be observed that the CV elimination rate decreases from 84% to 49% when the initial CV concentration varies from 20 to 200 mg/L. The decrease in the removal rate is probably due to the increase in the number of CV ions in the solution for the same number of sites and the same adsorbent surface area.

#### 3.3.3. Effect of the Initial pH of the Solution

pH is a critical parameter to be taken into account when removing dyes from aqueous solutions, as it can affect the charge on the surface of the adsorbent. The zero charge point pH_pzc_ of AS was 4.7 (inset in Figure 3(c)). The percentage of CV removal by the AS at different pH values was then studied, while keeping the other parameters at constant values. The results show that the highest removal efficiency of the CV (82%) was observed in the pH range of 6–12. This efficiency decreased to 62% at a pH of 2. Indeed, at pH > pH_pzc_, the surface of the AS is negatively charged, and this charge increases proportionally to the pH. Therefore, removal efficiency increases when the pH is in the range of 3–6 due to attractive forces occurring between the cationic dye and the negatively charged surface. Consequently, the optimal pH value that maximizes the removal of dye from the aqueous solution is 6.

#### 3.3.4. Effect of Adsorbent's Dose

CV adsorption on AS was studied by varying the dose of the adsorbent from 40 to 400 mg for a concentration of 30 mg/L of CV and a pH = 6. According to Figure 3(d), it can be seen that CV removal has increased rapidly from 42% to 82% in the range of 40–200 mg SA and remains constant in the range of 200 mg–400 mg; this is due to the increase in the contact surface, thus establishing the equilibrium. The adsorbent dose was set at 200 mg for the subsequent experiments.

### 3.4. Adsorption Equilibrium Study

The adsorption isotherm is the curve binding, at a fixed temperature, the quantity of product adsorbed per initial mass of adsorbent at the concentration residual in the solution after adsorption equilibrium. It was used to determine the maximum adsorption capacity and the type of interaction between the CV and AS. To exploit the data from the CV adsorption isotherm by AS, the Langmuir and Freundlich equations in their linear form were used. The linear form of the Langmuir isotherm is indicated in the following equation:

(3) $$\begin{array}{c} \frac{C_{e}}{q_{e}}=\frac{1}{K_{L}\cdot Q_{m}}+\frac{C_{e}}{Q_{m}}. \end{array}$$

The linear form of the Freundlich isotherm is indicated in the following equation:

(4) $$\begin{array}{c} Lnq_{e}=LnK_{F}+\frac{1}{n}LnC_{e}, \end{array}$$

where *C*_*e*_ (mg/L) is the equilibrium concentration; *Q*_*e*_ (mg/g) is the equilibrium adsorbed quantity; *Q*_*m*_ (mg/g) is the maximum adsorption capacity; *K*_*L*_ (L/mg) is the Langmuir constant; and *K*_*F*_ and *n* are the Freundlich constants.

**Figure 4:**
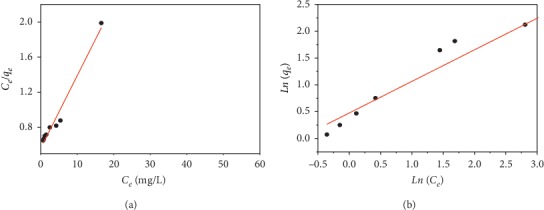
Adsorption isotherms of CV at the surface of AS: (a) Langmuir isotherms; (b) Freundlich isotherms.

The representations of the Langmuir and Freundlich models are given in Figure 4, and the equilibrium parameters obtained are shown in Table 3.

The value of the correlation coefficient (*R*^2^) for the Langmuir isotherm is higher than that of the Freundlich isotherm; this means that the Langmuir model better represents the adsorption process of CV by the AS. This suggests that CV fixation is done in monolayer, without interaction between the adsorbed molecules, on energetically equivalent sites. In addition to that, the *R*_*L*_ value is less than 1 which indicates that the adsorption of the dye is favorable.

### 3.5. Adsorption Kinetics

In order to model the adsorption kinetics, the kinetic models of the pseudo-first order and pseudo-second order were used. The expression of the pseudo-first-order model is in the form cited by Lagergren (5) [25]:

(5) $$\begin{array}{c} Ln\left(q_{e}-q_{t}\right)=Lnq_{e}-K_{1}t. \end{array}$$

The expression of the pseudo-second-order model is in the form cited by Ho and Mckay (6) [26]:

(6) $$\begin{array}{c} \frac{t}{q_{t}}=\frac{1}{K_{2}q_{e}^{2}}+\frac{1}{q_{e}}t, \end{array}$$

where *q*_*t*_ (mg·g^−1^) and *q*_*e*_ (mg·g^−1^) are the adsorbed quantity of dye at time *t* and at equilibrium and *k*_1_ (min^−1^) and *k*_2_ (g·mg^−1^·min) are the constants of pseudo-first-order and pseudo-second-order models, respectively.

**Figure 5:**
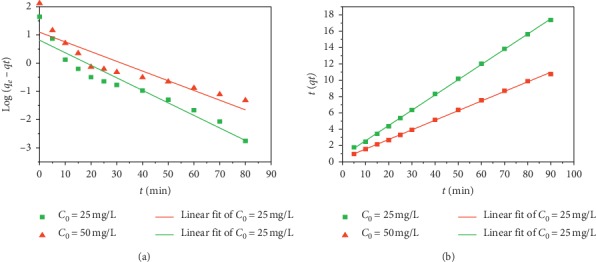
Kinetic adsorption of CV at the surface of AS: (a) pseudo-first-order kinetics; (b) Pseudo-second-order kinetics.

The curves of both models are shown in Figure 5, and the constants obtained from the different models are recapitulated in Table 4.

From the *R*^2^ values reported in Table 4, it can be deduced that the pseudo-second-order model is the one that best describes the CV adsorption process on AS. We also observe that the adsorbed quantities calculated by this model are closer to those determined experimentally.

### 3.6. Effect of Temperature and Thermodynamics of Adsorption

**Figure 6:**
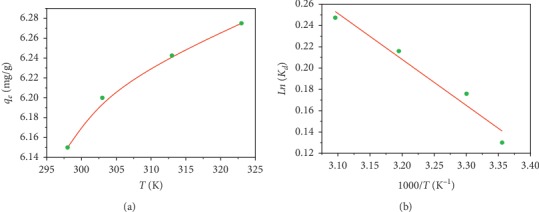
(a) Effect of temperature on malachite crystal violet rate; (b) plot ln (*K*_*d*_) *vs* 1/*T* for crystal violet adsorption into the almond shell.

The thermodynamic study was conducted at 25, 30, 40, and 50°C. The tests were performed on 40 ml mixtures of dye solutions at a concentration of 30 mg·L^−1^, with 160 mg masses of AS in 100 mL flasks. These mixtures were maintained at constant agitation of 200 rpm^−1^ for a time of 4 hours.  Figure 6(a) shows the influence of temperature on the dye retention rate. From the figure, we notice that this rate increases with increasing temperature, suggesting that the process is endothermic, and that increasing temperature promotes its progress.

Thermodynamic parameters such as standard Gibbs free energy change (Δ*G*°), standard enthalpy change (Δ*H*°), and standard entropy change (Δ*S*°) were determined by using the following equations [25]:

(7) $$\begin{array}{c} Ln\left(K_{d}\right)=\frac{\Delta {}^{S^{{}^{\circ}}}}{R}-\frac{\Delta {}^{H^{{}^{\circ}}}}{RT}, \end{array}$$

(8) $$\begin{array}{c} \Delta {}^{G^{{}^{\circ}}}=\Delta {}^{H^{{}^{\circ}}}-T\Delta {}^{S^{{}^{\circ}}}, \end{array}$$

where *K*_*d*_=*q*_*e*_/*C*_*e*_: distribution constant; *R*: universal gas constant (8.314 J/mol K); and *T*: absolute temperature (K).

Table 5 gives the values of standard Gibbs free energy change (Δ*G*°), standard enthalpy change (Δ*H*°), and standard entropy change (Δ*S*°), extrapolated from the plot ln (*K*_*d*_) *vs* 1/*T* (Figure 6(b)). The positive value of Δ*H*° shows that the adsorption process of the CV on AS is endothermic and that it is indeed a physisorption (<40 kJ·mol^−1^) [27].

The negative values of ∆*G*° indicate that the adsorption is spontaneous while the positive value of ∆*S*° indicates the increase in randomness at the solid-liquid interface during sorption. This is the normal consequence of the phenomenon of physical sorption, which occurs through electrostatic interactions. Similar results were obtained during the adsorption of malachite green by almond gum [28] as well as the adsorption of methylene blue by garlic straw [29].

### 3.7. Adsorption Mechanism

To understand the adsorption mechanism, it is necessary to examine the structure of the adsorbate and the properties of the adsorbent surface. For this purpose, it should be noted that CV is a cationic dye with amine groups in its structure and in aqueous medium dissociates into CV^+^ and Cl^−^ [30]. On the other hand, AS is a lignocellulosic material consisting of cellulose, hemicellulose, and lignin and other minor constituents [31, 32]. Cellulose and hemicellulose contain the majority of functional groupa, such as hydroxyl and carboxyl (confirmed by the FTIR spectrum), while lignin is a complex, systematically polymerized, and highly aromatic substance and acts as a cementing matrix that is maintained between and in both cellulose and hemicellulose units. In this study, the removal of CV by AS adsorption is highly pH dependent (Figure 3(c)). The CV has been adequately adsorbed for pH ≥ 5.

Based on the experimental results of this study, and depending on the structure of the adsorbate and the properties of the adsorbent surface, the mechanism for removing CV by AS adsorption involves the following steps:

- Migration of the dye from the solution to the surface of the adsorbent
- Dye diffusion through the boundary layer on the surface of the adsorbent
- Adsorption of the dye on the AS surface, which can be due to two mechanisms

**Figure 7:**
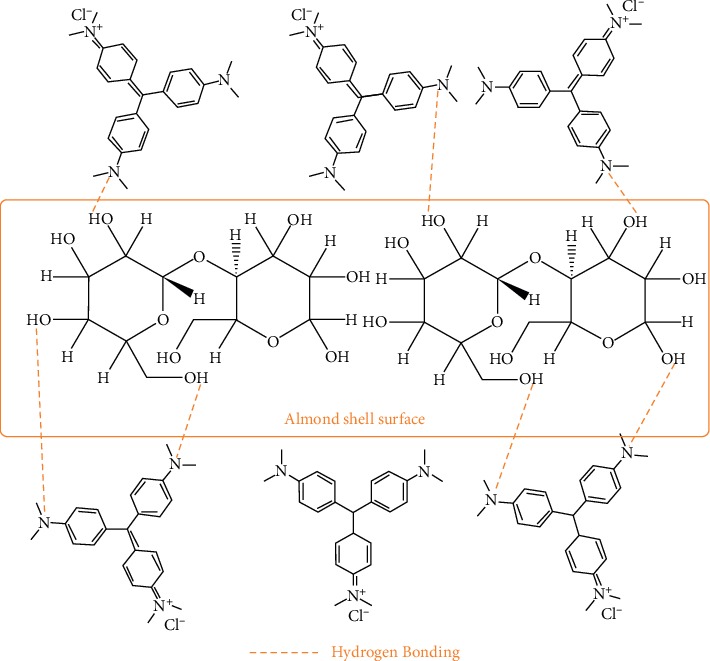
Schematic representation of hydrogen bonding between nitrogen atoms of CV and hydroxyl groups on the almond shell surface.

The first mechanism can explain the phenomenon of adsorption by the formation of hydrogen bonds between the surface hydroxyl and carboxyl groups and the nitrogen atoms of the CV as suggested in Figure 7.

**Figure 8:**
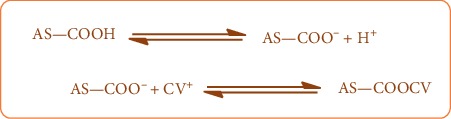
Schematic representation of dye-hydrogen ion exchange mechanism.

The second mechanism is a dye-hydrogen ion exchange mechanism because at pH ≥ 5, the surface functional groups are deprotonated and become negatively charged, which facilitates their binding to the positively charged CV molecules, as shown in Figure 8.

## 4. Conclusion

The almond shell, a low-cost and easily available material, has proven to be highly effective to remove crystal violet from aqueous solutions. The equilibrium data were analyzed using Langmuir and Freundlich isotherm models. The maximum monolayer adsorption capacity was equal to 12.2 mg/g. The experimental data of the adsorption isotherm follow the Langmuir model and the pseudo-second-order kinetic model. This work clearly shows that the elimination of crystal violet by the almond shell is feasible, efficient, and economical. Moreover, the almond shell is a promising candidate for wastewater treatment.

## Figures and Tables

**Table 1:** Adsorption capacities of some natural adsorbents for dye removal.

Adsorbents	Dye	Adsorption capacities (mg g^−1^)	References
Neem bark	Malachite green	0.36	[12]
Tamarind shell	Congo red	10.48	[13]
Grape fruit peel	Reactive blue 19	12.53	[14]
Peanut hull	Sunset yellow	13.99	[15]
Coir pith	Acid violet	1.6	[16]
Banana pith	Acid brilliant blue	4.42	[17]
Orange peel	Acid violet 17	19.88	[18]
Banana peel	Congo red	18.2	[19]
Corncob	Dye mixture	4.6	[20]
Potato peel	Methylene blue	33.55	[21]
Yellow passion fruit	Methylene blue	16.00	[22]

**Table 2:** Chemical properties and characteristics of crystal violet.

Generic name	Crystal violet
Chemical formula	C_25_H_30_N_3_Cl
Molecular weight (g/mol)	407.98
Type of dye	Cationic
*λ*_max_ (nm)	590 nm
Chemical structure	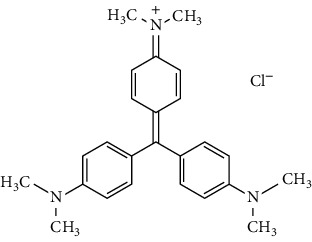

**Table 3:** Isotherm parameters for CV removal by AS.

Langmuir isotherm parameters	Freundlich isotherm parameters					
*Q*_*m*_ (cal.) (mg·g^−1^)	*R* ^2^	*K*_*L*_ (L·mg^−1^)	*R*_*L*_	*K*_f_ (L·g^−1^)	*n*	*R* ^2^
12.2	0.987	0.146	0.064	1.64	1.70	0.95807

**Table 4:** Pseudo-first-order and pseudo-second-order adsorption rate constants for the different initial CV concentrations (*C*_0_).

*C*_0_ (mg/L)	Pseudo-first-order kinetic			Pseudo-second-order kinetic		
	*q*_1_ (mg/g)	*K*_1_ (min^−1^)	*R* ^2^	*q*_2_ (mg/g)	*K*_2_ (g.mg^−1^min^−1^)	*R* ^2^
25	2.2557	0.04441	0.95606	5.3596	0.0471	0.99981
50	2.9961	0.0344	0.90404	8.5048	0.3399	0.99963

**Table 5:** Thermodynamic parameters for adsorption of crystal violet.

Température (K)	Δ*H*° (kJ·mol^−1^)	Δ*S*° (J·mol^−1^·K^−1^)	Δ*G*° (J·mol^−1^)	*R* ^2^
298	3.593	13.230	−349.54	0.980
303			−415.69	
313			−547.99	
323			−680.29	

## Data Availability

No data were used to support this study.
